# Ethyl 3-(2,4-difluoro­phen­oxy)-2-(4-methoxy­phen­yl)acrylate

**DOI:** 10.1107/S1600536808036945

**Published:** 2008-11-20

**Authors:** Wu Chen, Yong-Ming Cui, Fei Pan, Dong-Sheng Xia, Qing-Fu Zeng

**Affiliations:** a Engineering Research Center for Clean Production of Textile Printing, Ministry of Education, Wuhan University of Science & Engineering, Wuhan 430073, People’s Republic of China

## Abstract

In the title mol­ecule, C_18_H_16_F_2_O_4_, the two benzene rings form a dihedral angle of 55.2 (2)°. In the crystal structure, weak inter­molecular C—H⋯O hydrogen bonds link the mol­ecules into chains propagating along the *c* axis.

## Related literature

For a related crystal structure, see Fang *et al.* (2007 ▶). For the properties of phenyl­acetate and styrene derivatives, see: Huang *et al.* (2007 ▶); Li *et al.* (2007 ▶).
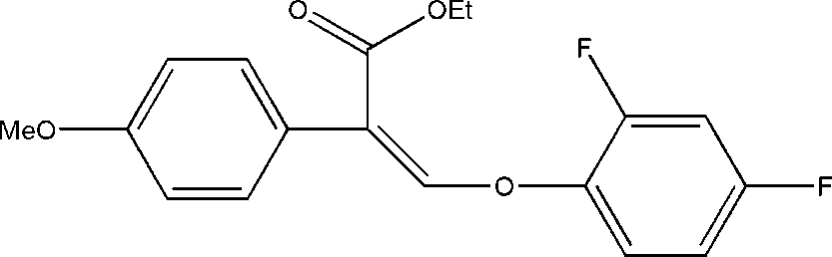


## Experimental

### 

#### Crystal data

C_18_H_16_F_2_O_4_
*M* *_r_* = 334.31
Monoclinic, 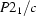

*a* = 17.295 (3) Å
*b* = 7.294 (2) Å
*c* = 14.233 (2) Å
β = 113.73 (3)°
*V* = 1643.7 (7) Å^3^
*Z* = 4
Mo *K*α radiation
μ = 0.11 mm^−1^
*T* = 298 (2) K
0.31 × 0.30 × 0.28 mm

#### Data collection

Bruker SMART 1000 CCD area-detector diffractometer
Absorption correction: multi-scan (*SADABS*; Bruker, 2001 ▶) *T*_min_ = 0.967, *T*_max_ = 0.970
3340 measured reflections
3198 independent reflections
1959 reflections with *I* > 2σ(*I*)
*R*_int_ = 0.017

#### Refinement

*R*[*F* ^2^ > 2σ(*F* ^2^)] = 0.062
*wR*(*F* ^2^) = 0.173
*S* = 1.03
3198 reflections
220 parameters
H-atom parameters constrained
Δρ_max_ = 0.43 e Å^−3^
Δρ_min_ = −0.31 e Å^−3^

### 

Data collection: *SMART* (Bruker, 2007 ▶); cell refinement: *SAINT* (Bruker, 2007 ▶); data reduction: *SAINT*; program(s) used to solve structure: *SHELXTL* (Sheldrick, 2008 ▶); program(s) used to refine structure: *SHELXTL*; molecular graphics: *SHELXTL*; software used to prepare material for publication: *SHELXTL*.

## Supplementary Material

Crystal structure: contains datablocks global, I. DOI: 10.1107/S1600536808036945/cv2469sup1.cif

Structure factors: contains datablocks I. DOI: 10.1107/S1600536808036945/cv2469Isup2.hkl

Additional supplementary materials:  crystallographic information; 3D view; checkCIF report

## Figures and Tables

**Table 1:** Hydrogen-bond geometry (Å, °)

*D*—H⋯*A*	*D*—H	H⋯*A*	*D*⋯*A*	*D*—H⋯*A*
C6—H6⋯O1^i^	0.93	2.52	3.280 (2)	140

Symmetry code: (i) 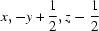.

**Fig. 1.**
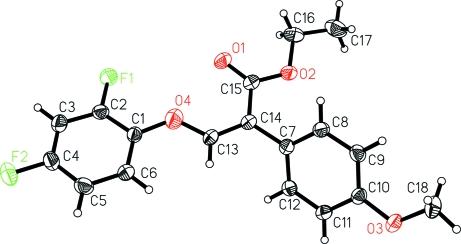
The molecular structure of (I), showing 30% probability displacement ellipsoids and the atom-numbering scheme.

## supplementary crystallographic information

### Comment

Phenylacetate and styrene derivatives are important in view of their extensive biological activities. Recently, many of such compounds with good activities were synthesized (Huang *et al.*, 2007; Li *et al.*, 2007). We report in this paper the title new compound, (I).

In (I) (Fig. 1), all bond lengths and angles are normal and correspond to those observed in the related compound (Fang *et al.*, 2007). The dihedral angles between C1—C6 and C7—C12 benzene rings is 55.2 (2)°. The O4/C13—C15/O1/O2 plane forms dihedral angles of 5.9 (2)° and 50.2 (2)°, respectively, with C1—C6 and C7—C12 benzene rings. In the crystal, weak intermolecular C—H···O hydrogen bonds (Table 1) link the molecules into chains propagated along *c* axis.

### Experimental

Ethyl 3-bromo-2-(2,4-difluorophenyl)acrylate (0.1 mmol) and 4-methoxyphenol (0.1 mmol) were reacted in chloroform (20 ml) for 12 h, giving a clear colorless solution. Crytals of the compound were formed by gradual evaporation of the solution.

### Refinement

All H atoms were placed in calculated positions with C–H = 0.93–0.97 Å, and refined as riding, with *U*_iso_(H) = 1.2–1.5*U*_eq_(C).

### Crystal data

**Table:**

C_18_H_16_F_2_O_4_	*F*_000_ = 696
*M**_r_* = 334.31	*D*_x_ = 1.351 Mg m^−^^3^
Monoclinic, *P*2_1_/*c*	Mo *K*α radiation λ = 0.71073 Å
Hall symbol: -P 2ybc	Cell parameters from 1232 reflections
*a* = 17.295 (3) Å	θ = 2.3–24.5º
*b* = 7.294 (2) Å	µ = 0.11 mm^−^^1^
*c* = 14.233 (2) Å	*T* = 298 (2) K
β = 113.73 (3)º	Block, colorless
*V* = 1643.7 (7) Å^3^	0.31 × 0.30 × 0.28 mm
*Z* = 4	

### Data collection

**Table:**

Bruker SMART 1000 CCD area-detector diffractometer	3198 independent reflections
Radiation source: fine-focus sealed tube	1959 reflections with *I* > 2σ(*I*)
Monochromator: graphite	*R*_int_ = 0.017
*T* = 298(2) K	θ_max_ = 26.0º
ω scans	θ_min_ = 1.3º
Absorption correction: multi-scan(SADABS; Bruker, 2001)	*h* = −21→19
*T*_min_ = 0.967, *T*_max_ = 0.970	*k* = −8→0
3340 measured reflections	*l* = 0→17

### Refinement

**Table:**

Refinement on *F*^2^	Hydrogen site location: inferred from neighbouring sites
Least-squares matrix: full	H-atom parameters constrained
*R*[*F*^2^ > 2σ(*F*^2^)] = 0.062	*w* = 1/[σ^2^(*F*_o_^2^) + (0.0671*P*)^2^ + 1.0598*P*] where *P* = (*F*_o_^2^ + 2*F*_c_^2^)/3
*wR*(*F*^2^) = 0.173	(Δ/σ)_max_ = 0.001
*S* = 1.03	Δρ_max_ = 0.43 e Å^−^^3^
3198 reflections	Δρ_min_ = −0.31 e Å^−^^3^
220 parameters	Extinction correction: SHELXTL (Sheldrick, 2008), Fc^*^=kFc[1+0.001xFc^2^λ^3^/sin(2θ)]^-1/4^
Primary atom site location: structure-invariant direct methods	Extinction coefficient: 0.021 (2)
Secondary atom site location: difference Fourier map	

### Special details

**Table:**

Geometry. All e.s.d.'s (except the e.s.d. in the dihedral angle between two l.s. planes) are estimated using the full covariance matrix. The cell e.s.d.'s are taken into account individually in the estimation of e.s.d.'s in distances, angles and torsion angles; correlations between e.s.d.'s in cell parameters are only used when they are defined by crystal symmetry. An approximate (isotropic) treatment of cell e.s.d.'s is used for estimating e.s.d.'s involving l.s. planes.
Refinement. Refinement of *F*^2^ against ALL reflections. The weighted *R*-factor *wR* and goodness of fit *S* are based on *F*^2^, conventional *R*-factors *R* are based on *F*, with *F* set to zero for negative *F*^2^. The threshold expression of *F*^2^ > σ(*F*^2^) is used only for calculating *R*-factors(gt) *etc*. and is not relevant to the choice of reflections for refinement. *R*-factors based on *F*^2^ are statistically about twice as large as those based on *F*, and *R*- factors based on ALL data will be even larger.

### Fractional atomic coordinates and isotropic or equivalent isotropic displacement parameters (Å^2^)

**Table:**

	*x*	*y*	*z*	*U*_iso_*/*U*_eq_	
F1	−0.10160 (10)	0.2759 (3)	0.53359 (13)	0.0659 (6)	
F2	−0.21582 (12)	0.0299 (3)	0.20055 (15)	0.0819 (7)	
O1	0.11843 (13)	0.3874 (4)	0.73642 (16)	0.0643 (7)	
O2	0.25578 (13)	0.4637 (3)	0.80903 (15)	0.0544 (6)	
O3	0.52673 (12)	0.3660 (3)	0.63131 (16)	0.0587 (6)	
O4	0.05420 (13)	0.2906 (4)	0.53932 (19)	0.0709 (7)	
C1	−0.01146 (16)	0.2255 (4)	0.4503 (2)	0.0429 (7)	
C2	−0.09183 (17)	0.2176 (4)	0.4484 (2)	0.0469 (7)	
C3	−0.16113 (18)	0.1544 (5)	0.3667 (2)	0.0565 (9)	
H3	−0.2144	0.1520	0.3682	0.068*	
C4	−0.14818 (19)	0.0949 (5)	0.2827 (2)	0.0562 (8)	
C5	−0.0706 (2)	0.0975 (5)	0.2794 (2)	0.0585 (9)	
H5	−0.0637	0.0558	0.2215	0.070*	
C6	−0.00211 (18)	0.1628 (5)	0.3632 (2)	0.0524 (8)	
H6	0.0510	0.1649	0.3612	0.063*	
C7	0.28913 (16)	0.3613 (4)	0.6413 (2)	0.0411 (7)	
C8	0.35658 (17)	0.2736 (4)	0.7170 (2)	0.0457 (7)	
H8	0.3479	0.2147	0.7699	0.055*	
C9	0.43628 (17)	0.2708 (4)	0.7167 (2)	0.0485 (8)	
H9	0.4803	0.2108	0.7686	0.058*	
C10	0.45029 (17)	0.3582 (4)	0.6383 (2)	0.0440 (7)	
C11	0.38402 (17)	0.4463 (4)	0.5619 (2)	0.0490 (8)	
H11	0.3928	0.5041	0.5087	0.059*	
C12	0.30513 (17)	0.4491 (4)	0.5639 (2)	0.0460 (7)	
H12	0.2615	0.5109	0.5124	0.055*	
C13	0.13631 (16)	0.3039 (4)	0.5529 (2)	0.0450 (7)	
H13	0.1485	0.2747	0.4967	0.054*	
C14	0.20244 (17)	0.3551 (4)	0.6398 (2)	0.0427 (7)	
C15	0.18686 (18)	0.4008 (4)	0.7308 (2)	0.0476 (7)	
C16	0.2474 (2)	0.5001 (6)	0.9039 (2)	0.0755 (11)	
H16A	0.2254	0.3927	0.9251	0.091*	
H16B	0.2085	0.6009	0.8950	0.091*	
C17	0.3315 (3)	0.5472 (6)	0.9827 (3)	0.0840 (12)	
H17A	0.3700	0.4482	0.9896	0.126*	
H17B	0.3273	0.5675	1.0472	0.126*	
H17C	0.3517	0.6565	0.9626	0.126*	
C18	0.59573 (18)	0.2753 (5)	0.7078 (3)	0.0696 (10)	
H18A	0.6035	0.3215	0.7741	0.104*	
H18B	0.6460	0.2967	0.6962	0.104*	
H18C	0.5846	0.1460	0.7049	0.104*	

### Atomic displacement parameters (Å^2^)

**Table:**

	*U*^11^	*U*^22^	*U*^33^	*U*^12^	*U*^13^	*U*^23^
F1	0.0494 (10)	0.1005 (16)	0.0561 (11)	0.0024 (10)	0.0299 (9)	−0.0084 (11)
F2	0.0591 (12)	0.1104 (18)	0.0587 (12)	−0.0114 (12)	0.0053 (10)	−0.0136 (12)
O1	0.0527 (13)	0.0918 (19)	0.0608 (14)	−0.0086 (12)	0.0358 (11)	−0.0089 (13)
O2	0.0562 (13)	0.0704 (16)	0.0429 (11)	−0.0079 (11)	0.0267 (10)	−0.0086 (11)
O3	0.0403 (11)	0.0748 (16)	0.0661 (14)	0.0039 (11)	0.0267 (10)	0.0059 (12)
O4	0.0547 (13)	0.0828 (18)	0.0761 (17)	−0.0016 (13)	0.0272 (12)	−0.0027 (14)
C1	0.0374 (14)	0.0455 (17)	0.0454 (16)	0.0002 (13)	0.0162 (13)	0.0024 (14)
C2	0.0463 (16)	0.0555 (19)	0.0440 (17)	0.0047 (14)	0.0235 (14)	0.0027 (15)
C3	0.0394 (16)	0.070 (2)	0.056 (2)	−0.0007 (16)	0.0150 (14)	0.0078 (18)
C4	0.0479 (18)	0.064 (2)	0.0462 (18)	−0.0024 (16)	0.0084 (15)	0.0012 (16)
C5	0.061 (2)	0.072 (2)	0.0460 (18)	0.0042 (18)	0.0242 (16)	−0.0044 (17)
C6	0.0447 (16)	0.067 (2)	0.0501 (18)	−0.0004 (15)	0.0239 (14)	−0.0033 (16)
C7	0.0401 (15)	0.0405 (17)	0.0440 (16)	−0.0023 (13)	0.0184 (13)	−0.0062 (13)
C8	0.0460 (16)	0.0496 (18)	0.0452 (16)	0.0030 (14)	0.0220 (13)	0.0043 (14)
C9	0.0431 (15)	0.053 (2)	0.0453 (17)	0.0089 (14)	0.0136 (13)	0.0044 (15)
C10	0.0392 (15)	0.0469 (18)	0.0484 (17)	0.0003 (13)	0.0201 (13)	−0.0052 (14)
C11	0.0462 (16)	0.058 (2)	0.0468 (17)	0.0006 (15)	0.0232 (14)	0.0080 (15)
C12	0.0409 (15)	0.0530 (19)	0.0424 (16)	0.0041 (14)	0.0151 (13)	0.0049 (15)
C13	0.0394 (15)	0.0490 (18)	0.0511 (17)	0.0018 (13)	0.0230 (13)	0.0003 (14)
C14	0.0417 (15)	0.0436 (17)	0.0470 (17)	−0.0008 (13)	0.0220 (13)	0.0003 (14)
C15	0.0500 (17)	0.0467 (18)	0.0510 (18)	−0.0026 (14)	0.0256 (15)	0.0012 (15)
C16	0.085 (3)	0.104 (3)	0.0470 (19)	−0.005 (2)	0.0371 (19)	−0.005 (2)
C17	0.109 (3)	0.090 (3)	0.047 (2)	−0.014 (3)	0.025 (2)	−0.006 (2)
C18	0.0424 (17)	0.086 (3)	0.077 (2)	0.0103 (18)	0.0202 (17)	0.003 (2)

### Geometric parameters (Å, °)

**Table:**

F1—C2	1.358 (3)	C8—C9	1.380 (4)
F2—C4	1.364 (3)	C8—H8	0.9300
O1—C15	1.222 (3)	C9—C10	1.388 (4)
O2—C15	1.343 (3)	C9—H9	0.9300
O2—C16	1.440 (4)	C10—C11	1.381 (4)
O3—C10	1.366 (3)	C11—C12	1.376 (4)
O3—C18	1.415 (4)	C11—H11	0.9300
O4—C13	1.357 (3)	C12—H12	0.9300
O4—C1	1.401 (3)	C13—C14	1.356 (4)
C1—C2	1.380 (4)	C13—H13	0.9300
C1—C6	1.391 (4)	C14—C15	1.464 (4)
C2—C3	1.370 (4)	C16—C17	1.475 (5)
C3—C4	1.373 (4)	C16—H16A	0.9700
C3—H3	0.9300	C16—H16B	0.9700
C4—C5	1.362 (4)	C17—H17A	0.9600
C5—C6	1.384 (4)	C17—H17B	0.9600
C5—H5	0.9300	C17—H17C	0.9600
C6—H6	0.9300	C18—H18A	0.9600
C7—C8	1.385 (4)	C18—H18B	0.9600
C7—C12	1.395 (4)	C18—H18C	0.9600
C7—C14	1.491 (4)		
			
C15—O2—C16	116.8 (2)	C12—C11—C10	120.4 (3)
C10—O3—C18	117.7 (2)	C12—C11—H11	119.8
C13—O4—C1	125.0 (2)	C10—C11—H11	119.8
C2—C1—C6	116.5 (3)	C11—C12—C7	121.5 (3)
C2—C1—O4	118.4 (3)	C11—C12—H12	119.2
C6—C1—O4	125.1 (2)	C7—C12—H12	119.2
F1—C2—C3	118.8 (3)	C14—C13—O4	126.9 (3)
F1—C2—C1	117.1 (3)	C14—C13—H13	116.6
C3—C2—C1	124.1 (3)	O4—C13—H13	116.6
C2—C3—C4	116.8 (3)	C13—C14—C15	118.8 (2)
C2—C3—H3	121.6	C13—C14—C7	119.7 (2)
C4—C3—H3	121.6	C15—C14—C7	121.5 (3)
C5—C4—F2	119.5 (3)	O1—C15—O2	122.2 (3)
C5—C4—C3	122.3 (3)	O1—C15—C14	124.6 (3)
F2—C4—C3	118.2 (3)	O2—C15—C14	113.2 (2)
C4—C5—C6	119.3 (3)	O2—C16—C17	108.4 (3)
C4—C5—H5	120.4	O2—C16—H16A	110.0
C6—C5—H5	120.4	C17—C16—H16A	110.0
C5—C6—C1	121.0 (3)	O2—C16—H16B	110.0
C5—C6—H6	119.5	C17—C16—H16B	110.0
C1—C6—H6	119.5	H16A—C16—H16B	108.4
C8—C7—C12	117.0 (2)	C16—C17—H17A	109.5
C8—C7—C14	121.6 (3)	C16—C17—H17B	109.5
C12—C7—C14	121.3 (3)	H17A—C17—H17B	109.5
C9—C8—C7	122.3 (3)	C16—C17—H17C	109.5
C9—C8—H8	118.9	H17A—C17—H17C	109.5
C7—C8—H8	118.9	H17B—C17—H17C	109.5
C8—C9—C10	119.6 (3)	O3—C18—H18A	109.5
C8—C9—H9	120.2	O3—C18—H18B	109.5
C10—C9—H9	120.2	H18A—C18—H18B	109.5
O3—C10—C11	116.3 (3)	O3—C18—H18C	109.5
O3—C10—C9	124.4 (3)	H18A—C18—H18C	109.5
C11—C10—C9	119.3 (3)	H18B—C18—H18C	109.5
			
C13—O4—C1—C2	179.1 (3)	C8—C9—C10—C11	−0.1 (5)
C13—O4—C1—C6	0.8 (5)	O3—C10—C11—C12	−178.6 (3)
C6—C1—C2—F1	179.1 (3)	C9—C10—C11—C12	0.6 (5)
O4—C1—C2—F1	0.6 (4)	C10—C11—C12—C7	−1.1 (5)
C6—C1—C2—C3	−0.6 (5)	C8—C7—C12—C11	1.0 (4)
O4—C1—C2—C3	−179.1 (3)	C14—C7—C12—C11	−176.3 (3)
F1—C2—C3—C4	−179.2 (3)	C1—O4—C13—C14	−175.5 (3)
C1—C2—C3—C4	0.4 (5)	O4—C13—C14—C15	0.6 (5)
C2—C3—C4—C5	0.0 (5)	O4—C13—C14—C7	179.7 (3)
C2—C3—C4—F2	179.5 (3)	C8—C7—C14—C13	−128.4 (3)
F2—C4—C5—C6	−179.7 (3)	C12—C7—C14—C13	48.7 (4)
C3—C4—C5—C6	−0.2 (6)	C8—C7—C14—C15	50.7 (4)
C4—C5—C6—C1	0.0 (5)	C12—C7—C14—C15	−132.1 (3)
C2—C1—C6—C5	0.4 (5)	C16—O2—C15—O1	5.3 (5)
O4—C1—C6—C5	178.7 (3)	C16—O2—C15—C14	−175.9 (3)
C12—C7—C8—C9	−0.4 (4)	C13—C14—C15—O1	3.9 (5)
C14—C7—C8—C9	176.8 (3)	C7—C14—C15—O1	−175.2 (3)
C7—C8—C9—C10	0.0 (5)	C13—C14—C15—O2	−174.9 (3)
C18—O3—C10—C11	−179.3 (3)	C7—C14—C15—O2	6.0 (4)
C18—O3—C10—C9	1.5 (4)	C15—O2—C16—C17	173.7 (3)
C8—C9—C10—O3	179.1 (3)		

### Hydrogen-bond geometry (Å, °)

**Table:**

*D*—H···*A*	*D*—H	H···*A*	*D*···*A*	*D*—H···*A*
C6—H6···O1^i^	0.93	2.52	3.280 (2)	140

Symmetry codes: (i) *x*, −*y*+1/2, *z*−1/2.
